# Lactate levels in emergency department patients across all causes of physiologic instability

**DOI:** 10.1186/cc12925

**Published:** 2013-11-05

**Authors:** Kimie Ødorf, Danielle Day, Simon Skibsted, Marie K Jessen, Nicolaj Duus, Nathan I Shapiro, Daniel Henning

**Affiliations:** 1 Research Center for Emergency Medicine, Aarhus University Hospital, Aarhus, Denmark; 2 Research Department of Emergency Medicine, Beth Israel Deaconess Medical Center and Harvard Medical School, Boston, MA, USA

## Background

Physiologic instability (PI) is a common, critical problem in the emergency department (ED) [1,2], and can have different underlying causes. The ability to determine the underlying cause of instability is paramount for early treatment and risk stratification [3]. Lactate has been shown to have prognostic value in some categories of unstable patients [4,5]. The objective of this study was to investigate how serum lactate concentrations differ across categories of PI and the association of lactate concentrations with clinical deterioration for each category.

## Materials and methods

A prospective observational study of adult patients with PI at a university ED. PI was defined as lactate ≥4 mmol/l, or >5 minutes of heart rate (HR) ≥130, or respiratory rate (RR) ≥24, or shock index ≥1, or systolic blood pressure ≤90 mmHg. We excluded patients with no lactate measurements, isolated atrial tachycardia, seizure, intoxication, psychiatric agitation, or tachycardia due to pain. A physician retrospectively categorized PI cause. Categories were defined as septic, cardiogenic, hemorrhagic, hypovolemic, or other. The primary outcome was deterioration, defined as: acute renal failure (elevated creatinine to ≥2× baseline levels), intubation, vasopressors, or in-hospital mortality.

## Results

**Figure 1:**
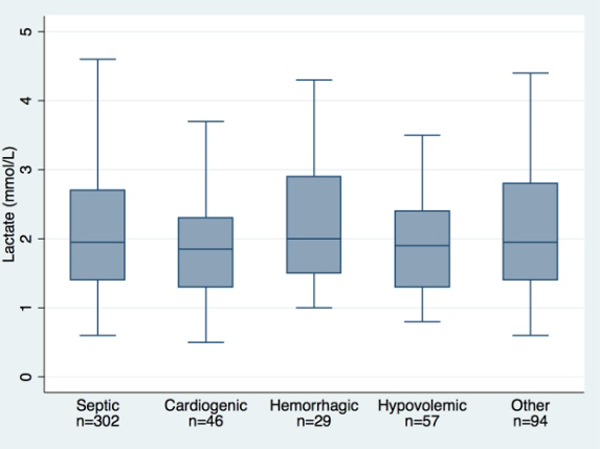
**Lactate levels across groups of physiological instability**.

**Figure 2:**
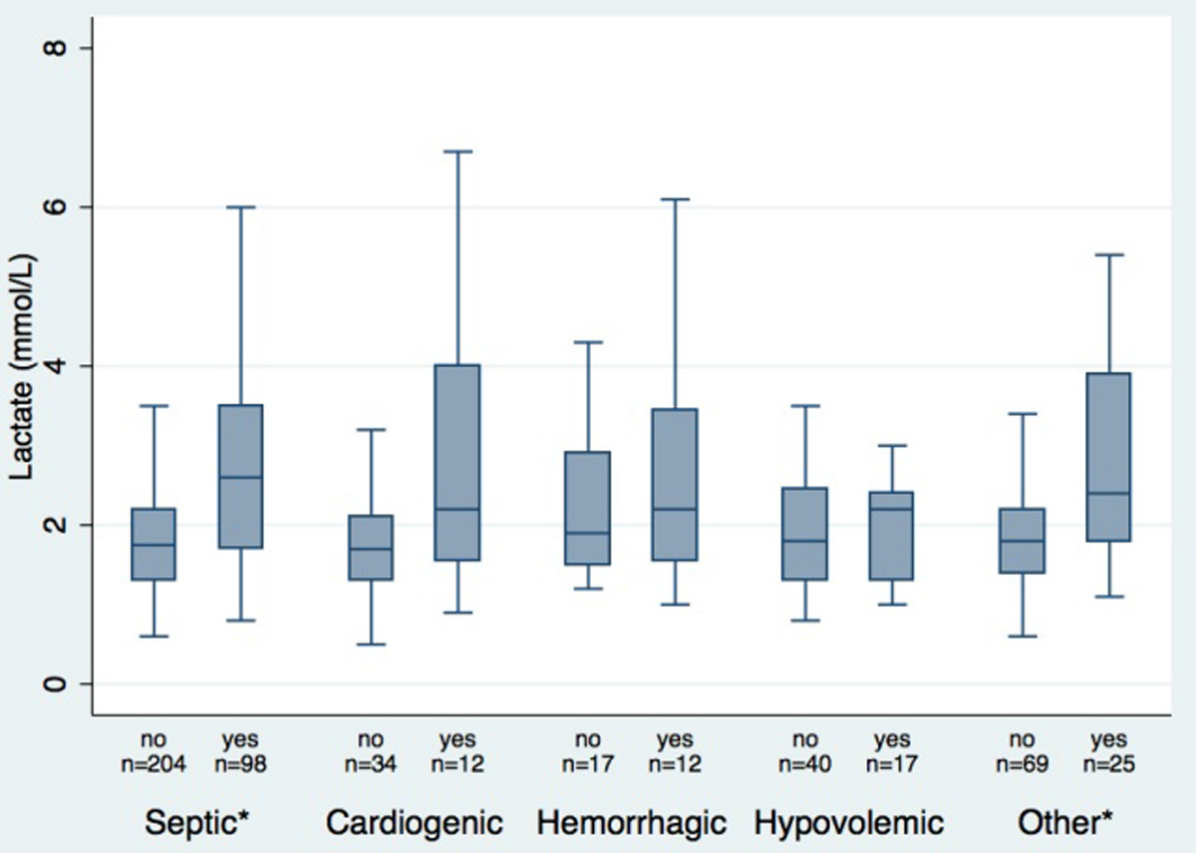
**Levels of lactate across groups of physiologic instability stratified by deterioration**. no/yes = deterioration present or not.(*) Significant differences between groups (*p *< 0.05).

We identified 1,156 patients with PI and excluded 324. Of the remaining, 304 did not have lactate measurements, leaving 528 for the analysis: 302 septic, 46 cardiogenic, 29 hemorrhagic, 57 hypovolemic, and 94 with another cause of instability. The differences in lactate levels between groups were not statistically significant (Figure 1). The lactate levels were statistically different between patients who deteriorated when compared with patients who did not deteriorate in the sepsis group (3.05 mmol/l vs. 1.91 mmol/l, *P *< 0.0001) and the other group (2.89 mmol/l vs. 1.94 mmol/l, *P *= 0.002). No statistically significant differences were demonstrated for the cardiogenic, the hemorrhagic or the hypovolemic groups (Figure 2).

## Conclusions

Lactate levels were not significantly different between the five groups with PI. However, in patients in the sepsis or other group, elevated lactate predicted deterioration. This was not demonstrated for the other causes of PI. This study suggests that in unstable patients lactate has the same likelihood of elevation between different causes of instability, but it may not have the same prognostic value for deterioration across underlying causes.

## References

[B1] Jones AE Aborn LS Kline JA Severity of emergency department hypotension predicts adverse hospital outcome Shock 2004 17 410 414 10.1097/01.shk.0000142186.95718.82 15489632

[B2] Jones AE Stiell IG Nesbitt LP Spaite DW Hasan N Watts BA Kline JA Nontraumatic out-of-hospital hypotension predicts inhospital mortality Ann Emerg Med 2004 17 106 113 10.1016/j.annemergmed.2003.08.008 14707949

[B3] Sebat F Musthafa Aa Johnson D Kramer Aa Shoffner D Eliason M Henry K Spurlock B Effect of a rapid response system for patients in shock on time to treatment and mortality during 5 years Crit Care Med 2007 17 2568 2575 10.1097/01.CCM.0000287593.54658.89 17901831

[B4] Shapiro NI Howell MD Talmor D Nathanson LA Wolfe RE Weiss JW Serum lactate as a predictor of mortality in emergency department patients with infection 2005 17 524 528 10.1016/j.annemergmed.2004.12.006 15855951

[B5] Vermeulen RP Hoekstra M Nijsten MW van der Horst IC van Pelt LJ Jessurun Ga Jaarsma T Zijlstra F van den Heuvel AF Clinical correlates of arterial lactate levels in patients with ST-segment elevation myocardial infarction at admission: a descriptive study Crit Care 2010 17 R164 10.1186/cc9253 20825687 PMC3219257

